# Preventing metabolic-associated fatty liver disease with fermented cordyceps preparation: an electronic medical record based study

**DOI:** 10.3389/fmed.2025.1576029

**Published:** 2025-05-14

**Authors:** Xiaozhou Zhou, Zijian Tian, Shaoyun Li, Ruifeng Jing, Ziqing Liu, Peng Wu, Jian Shao, Jie Bai, Rong Huang, Ying Pan, Kaixin Zhou

**Affiliations:** ^1^College of Life Sciences, University of Chinese Academy of Sciences, Beijing, China; ^2^Guangzhou National Laboratory, Guangzhou, Guangdong, China; ^3^National Laboratory of Biomacromolecules, Institute of Biophysics, Chinese Academy of Sciences (CAS), Beijing, China; ^4^Fifth People’s Hospital of Chongqing, Chongqing, China; ^5^College of Pharmacy and Tianjin Key Laboratory of Molecular Drug Research, State Key Laboratory of Medicinal Chemical Biology, Nankai University, Tianjin, China; ^6^Central Hospital Affiliated to Shandong First Medical University, Jinan, Shandong, China; ^7^Department of General Practice, Kunshan Hospital Affiliated to Jiangsu University, Kunshan, Jiangsu, China

**Keywords:** MAFLD, Fermented Cordyceps Preparation, metabolic syndrome, efficacy, safety

## Abstract

**Background:**

Metabolic-associated fatty liver disease (MAFLD) is a prevalent chronic liver condition with significant health implications. Fermented Cordyceps Preparation (FCP) has shown promise in managing metabolic disorders, prompting interest in its potential for MAFLD prevention. There is, however, a lack of large-scale clinical evidence regarding its preventive efficacy and long-term safety.

**Aim:**

We aimed to assess the preventive efficacy and safety of FCP, as regards combatting MAFLD.

**Methods:**

Propensity score matching was used to select 343 FCP users and 1372 non-users with metabolic syndrome, (MS) as recorded in EMR. These two groups were followed for 750 days, to track the incidence of MAFLD. The Kaplan Meier method was used to calculate the cumulative risk of MAFLD events in each subgroup. A Multiple linear regression model was used to compare the levels of alanine aminotransferase (ALT) and aspartate aminotransferase (AST), as between the two groups.

**Results:**

Compared with non-users, FCP users were associated with a 26% decreased risk of MAFLD (hazard ratio 0.74, 95% confidence interval 0.56–0.97). During the follow-up, the changes in both ALT and AST, were insignificantly different between the two groups.

**Conclusion:**

These findings highlight the potential of FCP in MAFLD prevention and offer insight into its safety profile, suggesting avenues for further clinical validation and drug repurposing efforts.

## Introduction

Metabolic-associated fatty liver disease (MAFLD) has become the most common chronic liver disease worldwide, with a prevalence rate as high as 32.4% (1). It not only significantly increases the risk of liver fibrosis but also the risks of complications such as cardiovascular diseases, cancer and even death (2), thus posing a significant medical and economic burden on patients and society (3). Prevention of MAFLD has, thus, become a major public health challenge.

Diabetes, obesity, dyslipidemia and insulin resistance, have been identified as risk factors for MAFLD (4). They are also the main components of metabolic syndrome (MS) (5). Clinical epidemiological studies have shown that MS is closely related to MAFLD (6). The incidence of MAFLD in patients with MS is as high as 73.1% (7). In a prospective cohort study, Japanese participants with MS were found to have up to an 11-fold increased risk of developing MAFLD in the future (8). Additionally, a long-term follow-up study in China also found that patients with MAFLD accompanied by metabolic disorders, had an increased mortality risk (9). Individuals with MS are, thus, the key MAFLD risk population in which to introduce early prevention.

International guidelines recommend that, the prevention and treatment of MAFLD should be based on personalized lifestyle interventions, in conjunction with active management of concomitant metabolic complications (10). Currently, however, there is no approved drug for the prevention and treatment of MAFLD (11).

Traditional Chinese medicine is widely used as complementary therapies in China (12). Cordyceps, mostly Fermented Cordyceps Preparation (FCP), is commonly used to support the management of renal function or lung function (13, 14). It has also recently emerged as a promising alternative therapy for metabolic diseases (15). Active ingredients such as cordycepin and polysaccharides in Cordyceps can exert long-term anti-inflammatory and anti-oxidant effects, which support the role of Cordyceps in combatting the aforementioned major drivers of MAFLD (16). Animal model studies also demonstrated that Cordyceps can inhibit fat accumulation in liver cells and prevent high-fat diet induced fatty liver (17). An elevated risk of liver damage was, however, also reported in some functional studies of Cordyceps (18). Prior studies showed great variability in Cordyceps dosages (19, 20). In contrast, FCP used in this study is a standardized Chinese patent medicine. Made from fermented Cordyceps fungus powder, it complies with pharmacopeia of the People’s Republic of China (2020). The pharmacopeia stipulates fixed dosages for different FCP products. Clinical studies of Cordyceps concerning MAFLD to date, have been fairly limited. One small study of 46 patients with hepatitis has shown that, Cordyceps can improve liver function and reduce fibrosis (21). Another study of 94 patients with fatty liver demonstrated significant improvement in lipid profile and the alleviating of liver inflammation, without causing significant liver toxicity (22, 23). These studies have, however, only examined the treatment potential of Cordyceps in small numbers of patients with established liver diseases over a short period. There is an urgent need to explore whether Cordyceps is suitable for MAFLD prevention in the high-risk group of individuals with MS.

Here, we report a large observational study of electronic medical records (EMR) that aims to explore the potential of using FCP to prevent MAFLD development. By following 1715 subjects with established MS for a period of over 2 years, we examined whether MAFLD incidence was reduced among FCP users, as compared to those without FCP intervention. Moreover, we also investigated whether long-term administration of FCP would pose a risk of liver injury. Our results are expected to offer preliminary evidence on the efficacy and safety of using FCP for the prevention of MAFLD.

## Materials and methods

### Study design and participants

This is a population based retrospective cohort study of EMR dating back to 2016. Participants were recruited between January 2019 and December 2022, when they underwent annual health examinations at the community health centers in the Kunshan county, Jiangsu Province, China. The 7-year data collection period, spanning from 2016, was crucial for identifying 15,406 individuals with metabolic syndrome (MS). This extended time frame not only provided a more extensive pool for subject selection but also facilitated a comprehensive analysis of data related to MS and subsequent follow - up.

All participants signed informed consent to contribute their EMR for research aimed at improving health care quality. Thus, we could identify subjects with multiple prescriptions of the Fermented Cordyceps Preparation (FCP), which is included in the Chinese Pharmacopeia and National Essential Medicines Catalog, and retrospectively monitored their biochemistry test records in EMR. The FCP used in this study is a standardized preparation made from fermented cordyceps fungus powder, produced according to the Pharmacopeia of the People’s Republic of China (2020) that specify raw material selection, fermentation conditions, and quality control for product consistency and quality. Details of the protocol for this study followed the guidelines of the Declaration of Helsinki and Tokyo for humans and approved by the institutional review board of the First People’s Hospital of Kunshan (IEC-C-007-A07-V3.0).

### Data source

For this study, EMRs were retrieved from three major databases via the integrated regional health informatics system that covered all aspects of the public health and medical care services managed by the local health commission.

The health examination database records the participants’ annual comprehensive physical examination results, including a large number of routine blood tests, B-ultrasound examination reports and lifestyle questionnaires. Dietary data, which are part of these lifestyle questionnaires, are collected during annual physicals. Doctors inquire about participants’ alcohol consumption, smoking, diet, and exercise. Participants report their daily food - related habits in response, and all this information is then recorded in the database. The chronic disease registry database records the incidence of common non-communicable diseases, including diabetes, hypertension, coronary artery disease, stroke and cancer. The regional medical information system, which provided access to outpatient prescription and diagnostic information, contained records of each patient’s prescribed FCP dosages as part of the prescription details. To ensure the reliability and comparability of the data, a series of pre-analysis procedures were implemented. Data from the three different databases (health examination database, chronic disease registry database, and regional medical information system) were cross-checked to identify and rectify any discrepancies. Standardization was also carried out, especially for continuous variables like laboratory test results. A unified reference range and calibration method were employed to minimize potential differences in measurement results. Additionally, strict quality control measures were implemented during the data collection and extraction processes to uphold the integrity of the data.

### Sample ascertainment

**FIGURE 1:**
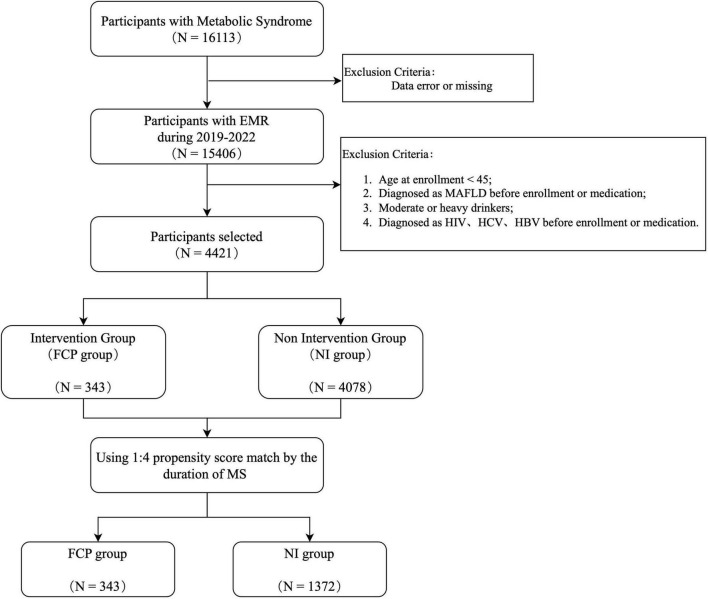
Sample ascertainment flow chart. It shows the process of participant selection starting from 16113 participants with Metabolic Syndrome. After exclusions due to data errors/missing and other specific criteria (such as age < 45, pre-enrollment diagnosis of MAPLD, malignancy, and certain viral infections), the remaining participants are divided into an Intervention Group (FCP group) and a Non - Intervention Group (NI group). A 1:4 propensity score match is then conducted based on the duration of MS.

To evaluate the health benefit of FCP in relation to MAFLD, we ascertained an intervention group (FCP group) and a non-intervention group (NI group), through the following procedure as shown in Figure 1. From the entire cohort, we identified 15,406 individuals with MS during a 7-year period. However, to ensure the homogeneity and relevance of the study groups, several exclusion criteria were applied. Individuals were excluded if they were aged under 45 at the time of the first FCP prescription (index date); had been reported with MAFLD prior to the index date; or with a historical record of moderate to significant alcohol consumption. Patients seropositive for HIV, hepatitis C, or B were excluded from the study to avoid potential confounding factors related to viral-induced liver and metabolic changes (24). A total number of 343 participants, who had received multiple FCP prescriptions were selected for the FCP group. The NI group was separated from the rest of the cohort at a 1:4 ratio using the propensity - score approach, based on the duration of MS and baseline covariates including variables closely related to MAFLD risk factors, such as age, gender, body mass index (BMI), fasting blood glucose (FBG), total cholesterol (TC), triglyceride (TG), high - density lipoprotein cholesterol (HDL-C), and low-density lipoprotein cholesterol (LDL-C). This method aimed to balance the potential confounding factors between the two groups, enhancing the validity of the comparison.

### Clinical variables

Baseline characteristics of the participants were defined as the last measurement prior to the index date. The follow-up time was defined as the time period from the index date of FCP exposure to the end of the observation. MS was defined according to the National Cholesterol Education Programme-Adult Treatment Panel III (NCEP-ATP III) (25), with the diagnostic indicators primarily coming from EMR and supplemented by information from the physical examination database. Incident MAFLD was captured by the ICD-10 codes of K76.x or B-ultrasound diagnostic report in the EMR. Alanine transaminase (ALT) and aspartate transaminase (AST) levels were used to assess the risk of liver injury. Based on the most recent annual physical examination records after enrollment, on average, there were no significant changes in participants’ overall dietary patterns and exercise levels.

### Statistical analysis

The characteristics of the participants were presented as the mean and standard deviation for quantitative parameters and percentages for categorical variables. Baseline characteristics of the FCP group and the NI group were compared using the ANOVA or chi-square test, as appropriate. The incidence rate of MAFLD was calculated by dividing the number of incident cases by the total follow-up period (person-years). The cumulative hazard of incident MAFLD events by each subgroup of participants was calculated using the Kaplan-Meier method, which was right censored at 750 days of the follow up. The association between baseline characteristics and MAFLD incidence was examined by multivariate Cox regression. The proportional hazard assumption in the Cox regression was assessed by use of the Schoenfeld residuals test.

A two-step analysis was used to elucidate the preventive effect of FCP. First, univariate and multivariate Cox regression were used to evaluate the independent contribution of each risk factor to the incidence of MAFLD in the FCP group and the NI group respectively. Then, significant risk factors were adjusted in the Kaplan-Meier survival analysis, to find out whether FCP exposure significantly inhibited the occurrence of MAFLD during the 750 days of observation. Whether the long-term use of FCP would cause elevated risk of liver injury was examined, by comparing the changes in ALT and AST between the two groups. All statistical analyses were performed using R studio software version 4.3.2. The statistical significance level was set at a 2-tailed *p* < 0.05.

## Results

### Baseline characteristics of the participants

After sample ascertainment, a total number of 343 FCP users with multiple prescriptions, were identified. From the remaining subjects, 1,372 non-users were selected by 1:4 propensity score matching, based on their duration of MS. Table 1 shows the median age of the participants was 68 years, with 63% being male. There is little significant difference between the FCP group and the NI group at baseline, except for higher blood urea nitrogen (BUN) and serum creatinine (Scr) in the FCP group, probably reflecting the common use of FCP to prevent or treat kidney diseases.

**TABLE 1:** Comparison of characteristics between NI group and FCP group.

Characteristics	NI group (*N* = 1372)	FCP group (*N* = 343)	*P*-value
**Demographic characteristics**			
Age, y	69.8 (9.11)	69.6 (8.88)	0.65
Male	872 (63.6%)	205 (59.8%)	0.20
BMI, kg/m^2^	24.5 (3.22)	24.2 (3.27)	0.16
**Clinical characteristics**			
SBP, mmHg	142 (18.8)	141 (20.9)	0.8
DBP, mmHg	80.6 (10.5)	80.3 (10.8)	0.66
FBG, mmol/L	6.81 (1.87)	6.67 (1.73)	0.17
TC, mmol/L	4.72 (1.02)	4.77 (1.04)	0.41
TG, mmol/L	1.87 (2.89)	1.90 (1.19)	0.78
HDL-C, mmol/L	1.32 (0.33)	1.33 (0.35)	0.94
LDL-C, mmol/L	2.63 (0.87)	2.66 (0.86)	0.58
TyG, (mg/dL)^2^	9.02 (0.60)	9.04 (0.61)	0.58
PLT,109/L	186 (57.5)	186 (55.6)	0.84
ALT, U/L	20.1 (13.0)	19.6 (21.1)	0.69
AST, U/L	21.8 (11.4)	21.5 (11.7)	0.64
BUN, mmol/L	5.93 (1.89)	6.88 (2.78)	<0.001
Scr, μmol/L	78.1 (37.0)	100 (90.6)	<0.001
**Lifestyle characteristics**			
Never smoked	954 (69.5%)	253 (73.8%)	0.14
Irregular exercise	815 (59.4%)	202 (58.9%)	0.91
Preferential diet	448 (32.7%)	115 (33.5%)	0.81

BMI, body mass index; SBP, systolic blood pressure; DBP, diastolic blood pressure; FBG, fasting blood glucose; TC, total cholesterol; TG, triglyceride; HDL-C, high-density lipoprotein cholesterol; LDL-C, low density lipoprotein; TyG, triglyceride glucose index; PLT, platelet; ALT, alanine transaminase; AST, aspartate transaminase; BUN, blood urea nitrogen; Scr, serum creatinine; preferential diet: in this study refers to an individual’s pronounced inclination toward certain types of foods, which may encompass preferences for foods that are high in fat, sugar, or salt.

### MAFLD risk profiling among the MS patients

All 1,715 participants were analyzed together, to comprehensively explore the MAFLD risk profile of these patients with MS. As shown in Table 2, univariable Cox regression analyses indicate being younger, overweight, hyperglycemic, dyslipidemic or insulin resistant, as assessed by high TyG, were all strongly (*p* < 0.001) associated with elevated MAFLD risk, in keeping with previous reports (26, 27). When these risk factors were examined together in multivariable Cox regression, only overweight and insulin resistance were independent risk factors for MAFLD (*p* < 0.001), hence, they were adjusted as covariates in the following investigations.

**TABLE 2:** Clinical factors contributing to the risk of MAFLD.

Factors	Univariable		Multivariable	
	HR (95% CI)	*p*.value	HR (95% CI)	*p*.value
**Demographic characteristics**				
Age	0.97 (0.96–0.99)	<0.001	0.99 (0.98–1.00)	0.143
Female	1.28 (1.04–1.57)	0.019	0.86 (0.64–1.15)	0.298
BMI	1.08 (1.05–1.10)	<0.001	1.05 (1.02–1.08)	<0.001
**Clinical characteristics**				
SBP	1.09 (0.98–1.20)	0.098	1.06 (0.94–1.20)	0.340
DBP	1.13 (1.03–1.25)	0.014	1.01 (0.89–1.14)	0.923
FBG	1.15 (1.06–1.26)	0.001	0.91 (0.80–1.04)	0.165
TC	1.20 (1.09–1.32)	<0.001	0.71 (0.50–1.01)	0.059
TG	1.09 (1.05–1.13)	<0.001	0.96 (0.89–1.03)	0.275
HDL-C	0.80 (0.72–0.89)	<0.001	1.04 (0.87–1.23)	0.694
LDL-C	1.20 (1.08–1.32)	<0.001	1.51 (1.13–2.01)	0.005
TyG	1.44 (1.31–1.58)	<0.001	1.65 (1.30–2.11)	<0.001
PLT	1.13 (1.02–1.24)	0.014	1.03 (0.93–1.14)	0.570
ALT	1.07 (1.01–1.14)	0.019	1.29 (1.09–1.52)	0.003
AST	0.94 (0.83–1.07)	0.373	0.73 (0.57–0.93)	0.011
**Lifestyle characteristics**				
Never smoked	1.15 (0.92–1.45)	0.222	1.07 (0.81–1.40)	0.647
Irregular exercise	0.96 (0.78–1.18)	0.702	0.99 (0.80–1.21)	0.903
Preferential diet	1.29 (1.05–1.59)	0.016	1.13 (0.91–1.39)	0.279

### The FCP group had a lower risk of MAFLD than the NI group

**FIGURE 2:**
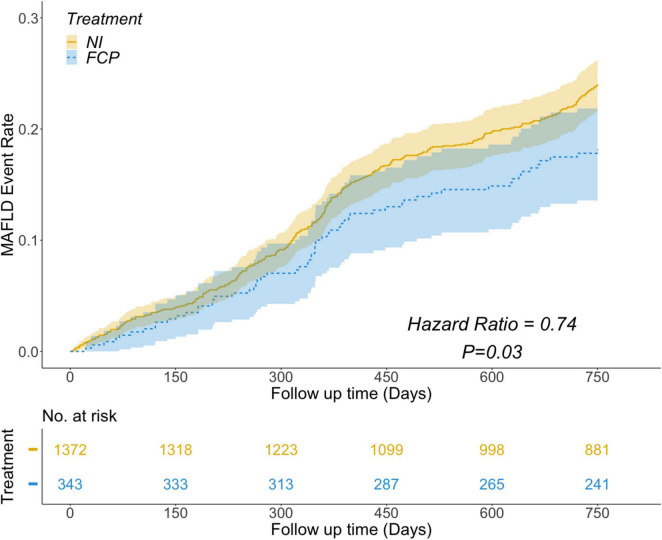
Results showing the risk of MAFLD event rate over follow - up time. The FCP group (blue) had a lower risk of MAFLD than the NI group (yellow), with a Hazard Ratio of 0.74 and a *P* - value of 0.03. The number at risk for both groups is shown at different follow - up time points.

Among the 1715 participants with MS, 376 (22.1%) experienced MAFLD during the follow-up period of 750 days. To explore the potential relationship between FCP treatment and MAFLD occurrence, a multivariable Cox regression analysis was performed. In the Kaplan-Meier survival analysis, adjustments were made for significant multivariable Cox regression factors (BMI, TyG, LDL-C, ALT, AST) and other important factors (FBG, TC, TG, HDLC, Preferential Diet) based on their roles in MS and MAFLD and univariate analysis results. As shown in Figure 2, after adjusting for relevant covariates, the analysis indicated a significant negative association. Specifically, individuals in the FCP group exhibited a 26% lower relative risk (HR = 0.74, *P* = 0.03) of developing MAFLD compared to those in the NI group.

### The FCP group and NI group had similar changes in ALT and AST levels

**FIGURE 3:**
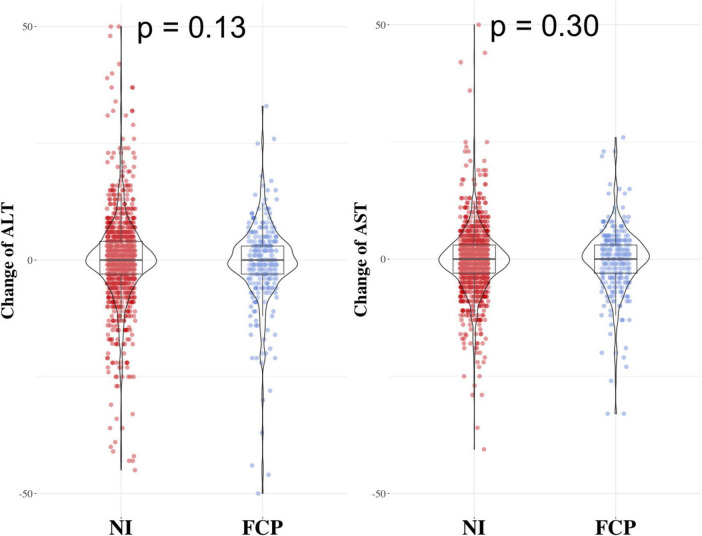
Results comparing the change in ALT and AST levels between the FCP group and the NI group. The two groups had similar changes in ALT (left side) and AST (right side) levels, with *p* - values of 0.13 for ALT and 0.30 for AST.

To investigate whether long-term prescribing of Cordyceps had an impact on the risk of liver injury, we compared the changes of two key indices of ALT and AST, between the two groups. As shown in Figure 3, the changes of neither ALT nor AST were significantly different between the NI group and the FCP group, after a median follow up duration of 307 days. These findings highlight the potential of FCP in MAFLD prevention and offer insight into its safety profile. Long - term administration of FCP did not significantly exacerbate the risk of liver damage estimated through AST and ALT levels.

## Discussion

This study leveraged a sizeable observational cohort, to explore the preventive potential of FCP against MAFLD in patients with MS, as well as the safety of long-term administration. Initially, through Kaplan-Meier survival analysis, we established that FCP use in MS patients significantly reduced the incidence of MAFLD. Additionally, our investigation revealed that, prolonged FCP administration in MS patients did not induce an extra risk of liver damage. These findings underscore the promise of FCP in drug repurposing, furnish robust evidence regarding their safety in the population and offer crucial clinical implications for MAFLD prevention strategies.

The primary focus of this study was to assess the preventive efficacy of FCP against MAFLD. While one previous investigation has demonstrated the therapeutic potential of FCP in reducing liver fat content in patients with established MAFLD (22), scant attention has been given to their preventive effects on individuals at high risk of developing MAFLD. To bridge this gap, a total of 1,715 participants with MS but without MAFLD were identified from the electronic medical records. These participants had an elevated risk of MAFLD owing to their existing condition of MS. These individuals were followed up through the data in the electronic medical records for an average of 1.7 years. We presented the first evidence that FCP treatment significantly decreased the incidence of MAFLD in MS patients. Our findings underscore the preventive potential of FCP against MAFLD and offer fresh insight into their repurposing potential as pharmaceutical agents.

Evidence surrounding the safety of long-term FCP administration has been conflicting (28), leaving a dearth of solid evidence on potential side effects in large-scale populations. Our study found no evidence of liver inflammation resulting from long-term FCP administration, furnishing crucial evidence for its safety and bridging the gap in large-scale population observations of FCP safety.

Although the pathogenesis of MAFLD remains not fully understood, evidence from preclinical models suggests that inflammation, oxidative stress and insulin resistance, are key drivers of disease progression (16). Recent studies have highlighted that, FCP contains bioactive compounds such as cordyceps acid (29), cordyceps polysaccharide (30) and ergosterol (31), all of which exhibit potent antioxidant and anti-inflammatory properties and can ameliorate insulin resistance. These components possess the ability to neutralize reactive oxygen species, inhibit pro-inflammatory mediators and improve hepatic insulin sensitivity. Thus, the regulation of inflammation, oxidative stress and insulin resistance, represents a potential molecular mechanism by which FCP exerts its preventive effect on MAFLD.

This study benefited from a large population cohort linked to the EMR system. The large number of participants in the EMR system allowed the identification of sufficient FCP users for investigation. The integrated regional EMR system also supported a 2-year follow up of many individuals, which may be challenging for clinical trials. Most importantly, comprehensive EMR data dating back to 2016 facilitated an in-depth exploration of clinical indicators potentially affecting the efficacy of FCPs in preventing MAFLD. This study could, thus, serve as a prime example of EMR-based large cohort studies in TCM investigations.

Given the observational nature of this study, several limitations merit acknowledgment. Firstly, the propensity score matching may not offer an ideal comparator NI group. Efforts were made to ensure no disparity in the time from MS diagnosis to enrollment between the FCP group and the NI group, aiming to establish similar metabolic disorder statuses at baseline. Secondly, although FCP dosage was recorded in the database as part of prescription details, assessing FCP exposure was tough. FCP being over - the - counter and the complex data format made it hard to figure out how FCP related to other medications when multiple prescriptions and diagnoses were present on 1 day. Dosage and indications in the EMR were not always clear. In relation to diet, dietary data from the database were used in multivariable models. However, the general advice and self-reported data (prone to recall bias) pose challenges to accurate dietary variable adjustment. Additionally, conventional drugs used by participants were not considered in this study. Nonetheless, since FCPs are primarily indicated for chronic kidney disease, the confounding caused by drugs used for the management of kidney disease is expected to be minimal. Despite these limitations, the results of this study suggest an association between FCP treatment and a reduced risk of MAFLD in MS patients. This finding underscores the need for further research, preferably well - controlled prospective studies, to firmly establish the nature of this relationship and to explore the underlying mechanisms.

## Conclusion

In conclusion, this observational study suggests a potential preventive effect of FCPs against MAFLD, as indicated by the negative association observed and reassures that long-term administration does not significantly exacerbate the risk of liver damage. These findings provide grounds for further trials to validate the clinical efficacy and safety of FCP, illustrating their broad prospects and potential value in drug repurposing endeavors.

## Data Availability

The raw data supporting the conclusions of this article will be made available by the authors, without undue reservation.
